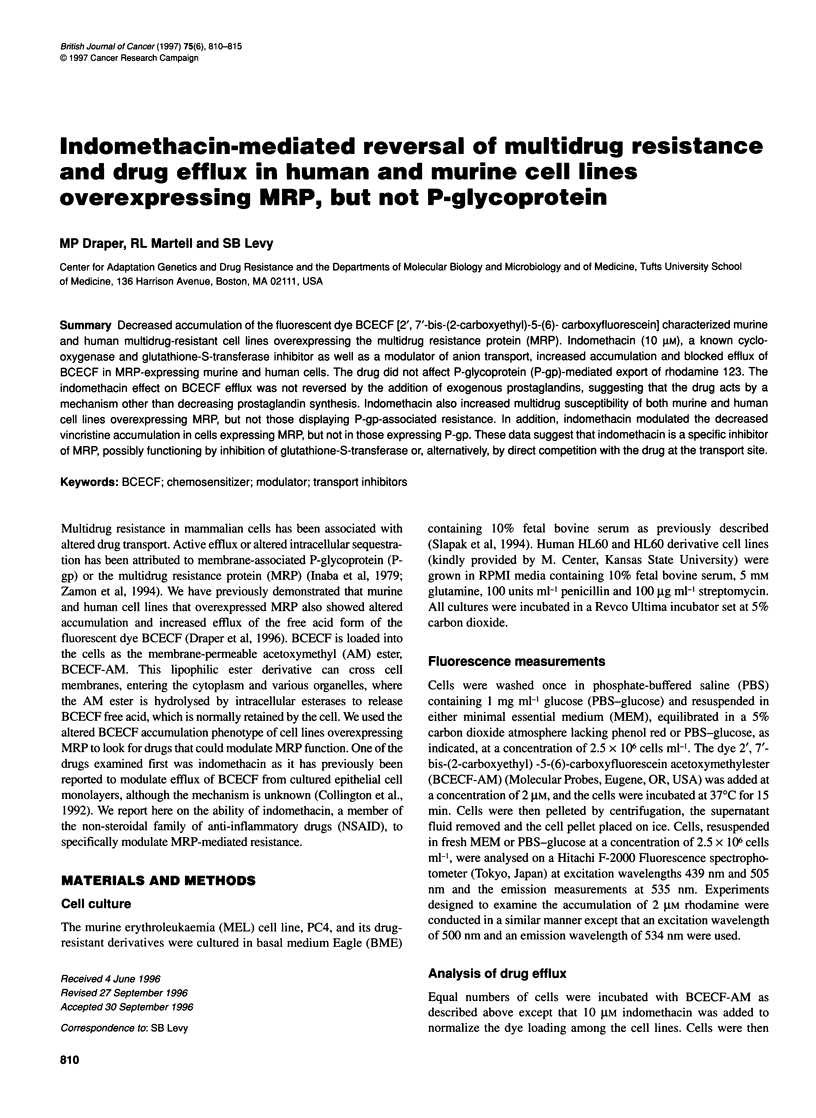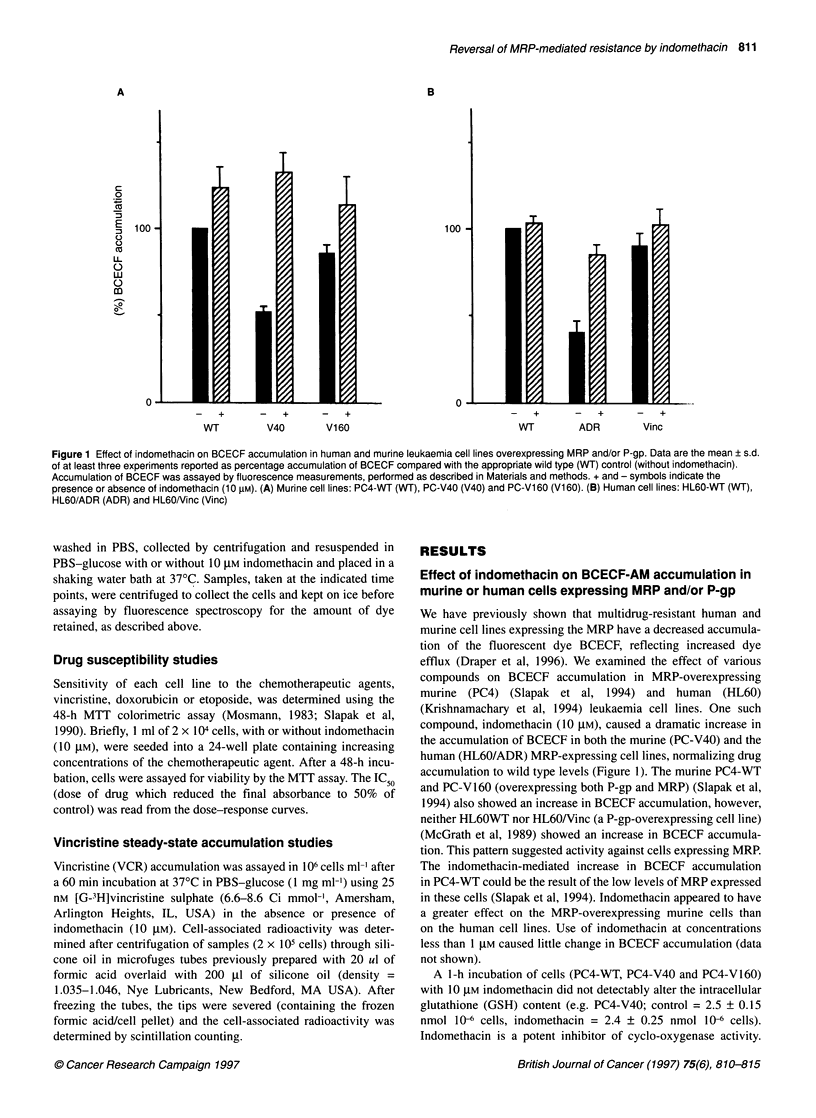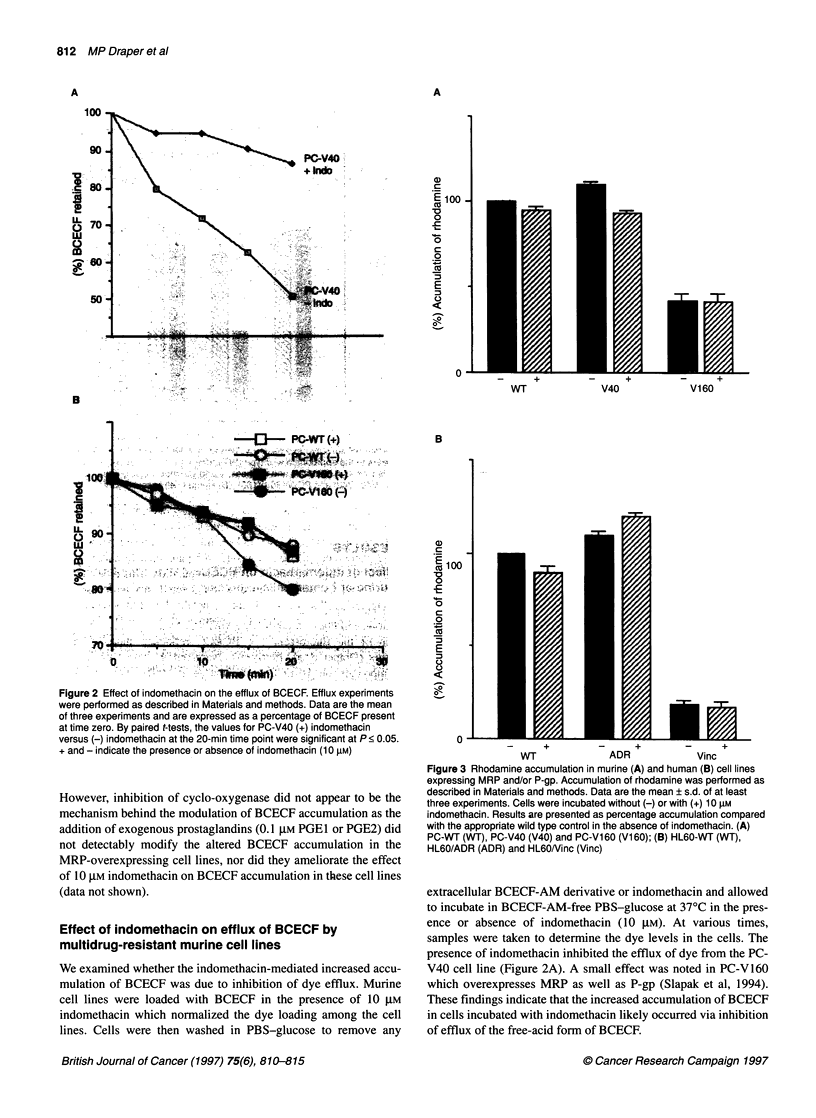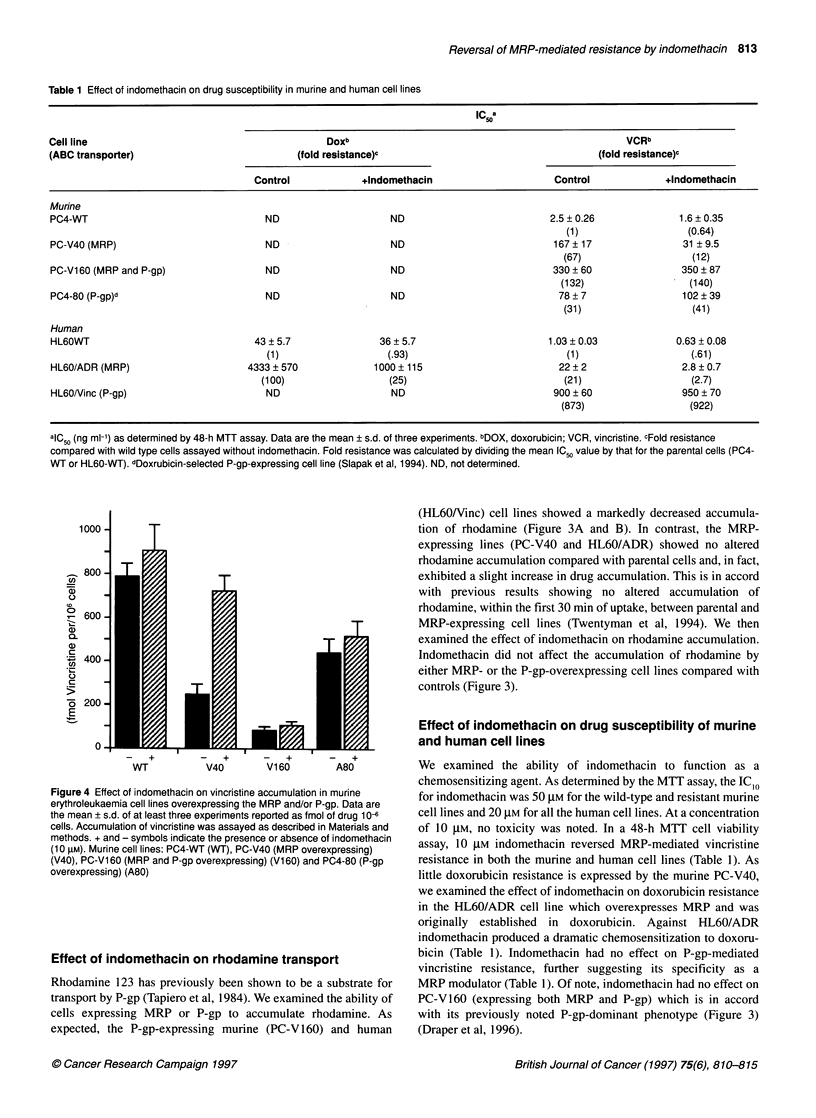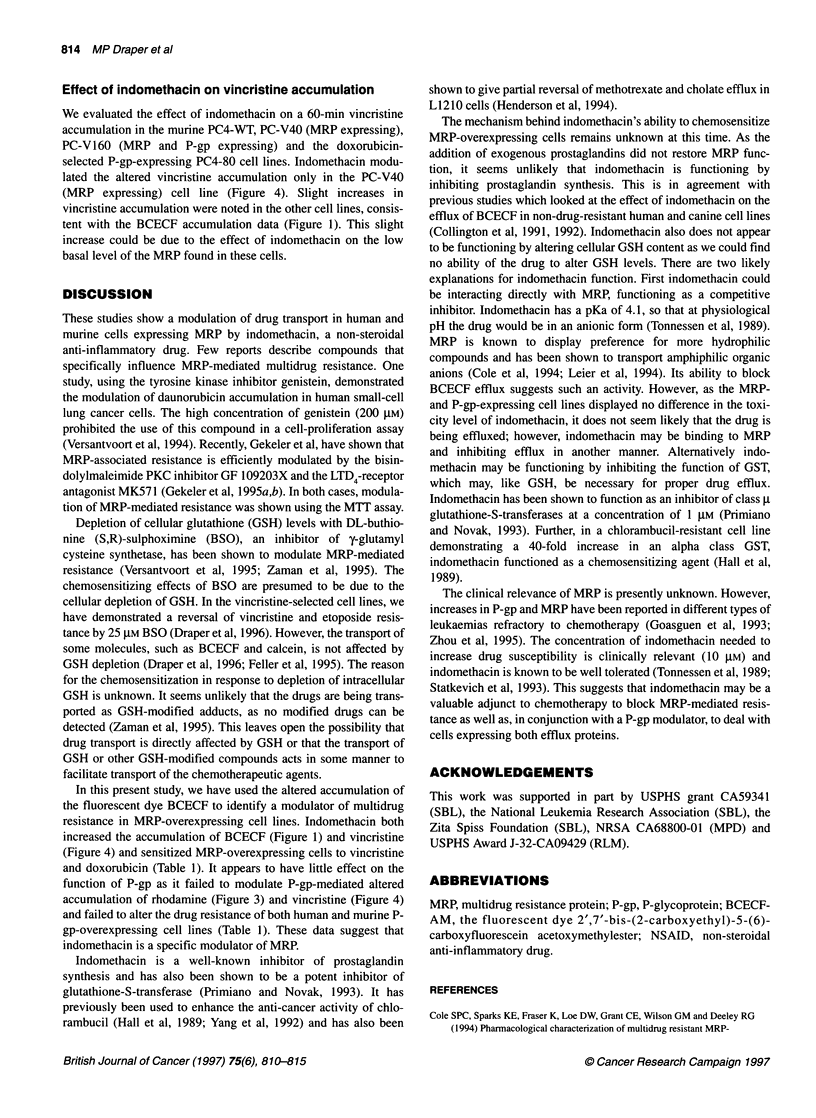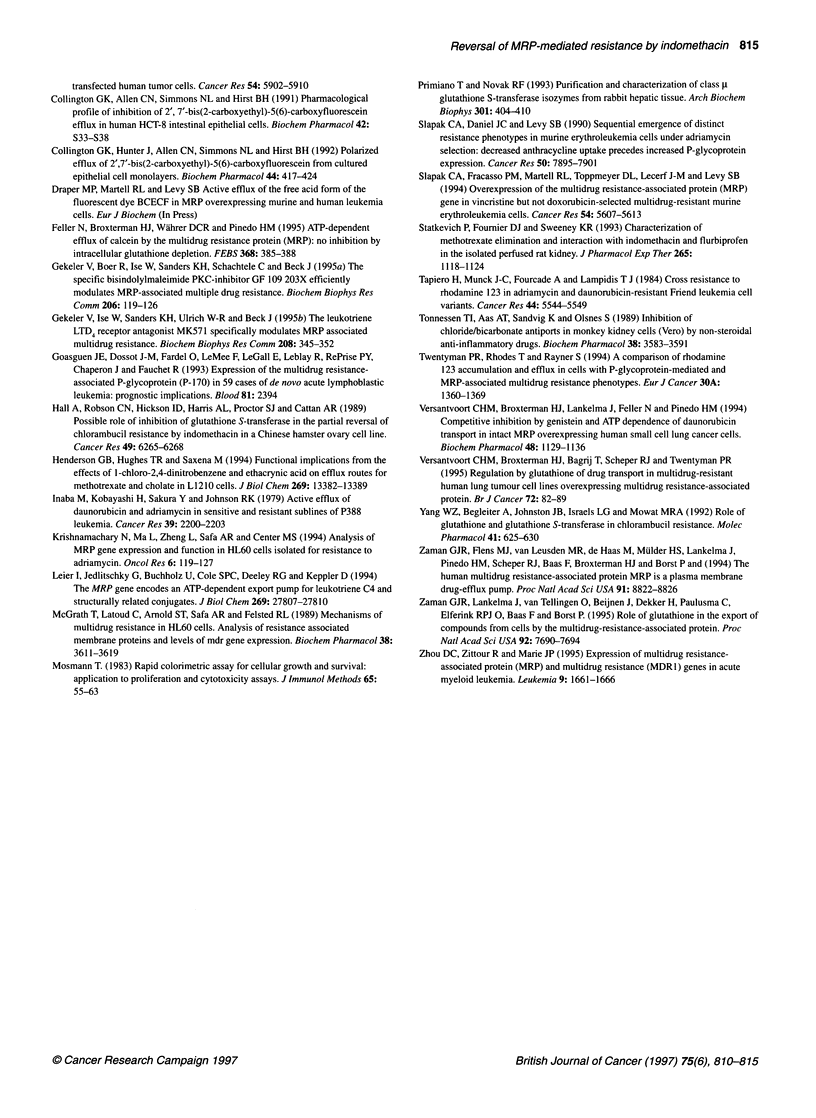# Indomethacin-mediated reversal of multidrug resistance and drug efflux in human and murine cell lines overexpressing MRP, but not P-glycoprotein.

**DOI:** 10.1038/bjc.1997.145

**Published:** 1997

**Authors:** M. P. Draper, R. L. Martell, S. B. Levy

**Affiliations:** Center for Adaptation Genetics and Drug Resistance and the Department of Molecular Biology, Tufts University School of Medicine, Boston, MA 02111, USA.

## Abstract

Decreased accumulation of the fluorescent dye BCECF [2', 7'-bis-(2-carboxyethyl)-5-(6)- carboxyfluorescein] characterized murine and human multidrug-resistant cell lines overexpressing the multidrug resistance protein (MRP). Indomethacin (10 microM), a known cyclo-oxygenase and glutathione-S-transferase inhibitor as well as a modulator of anion transport, increased accumulation and blocked efflux of BCECF in MRP-expressing murine and human cells. The drug did not affect P-glycoprotein (P-gp)-mediated export of rhodamine 123. The indomethacin effect on BCECF efflux was not reversed by the addition of exogenous prostaglandins, suggesting that the drug acts by a mechanism other than decreasing prostaglandin synthesis. Indomethacin also increased multidrug susceptibility of both murine and human cell lines overexpressing MRP, but not those displaying P-gp-associated resistance. In addition, indomethacin modulated the decreased vincristine accumulation in cells expressing MRP, but not in those expressing P-gp. These data suggest that indomethacin is a specific inhibitor of MRP, possibly functioning by inhibition of glutathione-S-transferase or, alternatively, by direct competition with the drug at the transport site.


British Journal of Cancer (1997) 75(6), 810–815
© 1997 Cancer Research Campaign

### Indomethacin-mediated reversal of multidrug resistance and drug efflux in human and murine cell lines overexpressing MRP, but not P-glycoprotein

**MP Draper, RL Martell and SB Levy**

Center for Adaptation Genetics and Drug Resistance and the Departments of Molecular Biology and Microbiology and of Medicine, Tufts University School of Medicine, 136 Harrison Avenue, Boston, MA 02111, USA

**Summary** Decreased accumulation of the fluorescent dye BCECF [2′, 7′-bis-(2-carboxyethyl)-5-(6)- carboxyfluorescein] characterized murine and human multidrug-resistant cell lines overexpressing the multidrug resistance protein (MRP). Indomethacin (10 μM), a known cyclo-oxygenase and glutathione-S-transferase inhibitor as well as a modulator of anion transport, increased accumulation and blocked efflux of BCECF in MRP-expressing murine and human cells. The drug did not affect P-glycoprotein (P-gp)-mediated export of rhodamine 123. The indomethacin effect on BCECF efflux was not reversed by the addition of exogenous prostaglandins, suggesting that the drug acts by a mechanism other than decreasing prostaglandin synthesis. Indomethacin also increased multidrug susceptibility of both murine and human cell lines overexpressing MRP, but not those displaying P-gp-associated resistance. In addition, indomethacin modulated the decreased vincristine accumulation in cells expressing MRP, but not in those expressing P-gp. These data suggest that indomethacin is a specific inhibitor of MRP, possibly functioning by inhibition of glutathione-S-transferase or, alternatively, by direct competition with the drug at the transport site.

**Keywords:** BCECF; chemosensitizer; modulator; transport inhibitors

Multidrug resistance in mammalian cells has been associated with altered drug transport. Active efflux or altered intracellular sequestration has been attributed to membrane-associated P-glycoprotein (P-gp) or the multidrug resistance protein (MRP) (Inaba et al, 1979; Zamon et al, 1994). We have previously demonstrated that murine and human cell lines that overexpressed MRP also showed altered accumulation and increased efflux of the free acid form of the fluorescent dye BCECF (Draper et al, 1996). BCECF is loaded into the cells as the membrane-permeable acetoxymethyl (AM) ester, BCECF-AM. This lipophilic ester derivative can cross cell membranes, entering the cytoplasm and various organelles, where the AM ester is hydrolysed by intracellular esterases to release BCECF free acid, which is normally retained by the cell. We used the altered BCECF accumulation phenotype of cell lines overexpressing MRP to look for drugs that could modulate MRP function. One of the drugs examined first was indomethacin as it has previously been reported to modulate efflux of BCECF from cultured epithelial cell monolayers, although the mechanism is unknown (Collington et al., 1992). We report here on the ability of indomethacin, a member of the non-steroidal family of anti-inflammatory drugs (NSAID), to specifically modulate MRP-mediated resistance.

### MATERIALS AND METHODS

### Cell culture

The murine erythroleukaemia (MEL) cell line, PC4, and its drug-resistant derivatives were cultured in basal medium Eagle (BME) containing 10% fetal bovine serum as previously described (Slapak et al, 1994). Human HL60 and HL60 derivative cell lines (kindly provided by M. Center, Kansas State University) were grown in RPMI media containing 10% fetal bovine serum, 5 mM glutamine, 100 units ml$^{-1}$ penicillin and 100 μg ml$^{-1}$ streptomycin. All cultures were incubated in a Revco Ultima incubator set at 5% carbon dioxide.

### Fluorescence measurements

Cells were washed once in phosphate-buffered saline (PBS) containing 1 mg ml$^{-1}$ glucose (PBS–glucose) and resuspended in either minimal essential medium (MEM), equilibrated in a 5% carbon dioxide atmosphere lacking phenol red or PBS–glucose, as indicated, at a concentration of $2.5 \times 10^6$ cells ml$^{-1}$. The dye 2′, 7′-bis-(2-carboxyethyl) -5-(6)-carboxyfluorescein acetoxymethylester (BCECF-AM) (Molecular Probes, Eugene, OR, USA) was added at a concentration of 2 μM, and the cells were incubated at 37°C for 15 min. Cells were then pelleted by centrifugation, the supernatant fluid removed and the cell pellet placed on ice. Cells, resuspended in fresh MEM or PBS–glucose at a concentration of $2.5 \times 10^6$ cells ml$^{-1}$, were analysed on a Hitachi F-2000 Fluorescence spectrophotometer (Tokyo, Japan) at excitation wavelengths 439 nm and 505 nm and the emission measurements at 535 nm. Experiments designed to examine the accumulation of 2 μM rhodamine were conducted in a similar manner except that an excitation wavelength of 500 nm and an emission wavelength of 534 nm were used.

### Analysis of drug efflux

Equal numbers of cells were incubated with BCECF-AM as described above except that 10 μM indomethacin was added to normalize the dye loading among the cell lines. Cells were then

Received 4 June 1996
Revised 27 September 1996
Accepted 30 September 1996

Correspondence to: SB Levy

**Figure 1** Effect of indomethacin on BCECF accumulation in human and murine leukaemia cell lines overexpressing MRP and/or P-gp. Data are the mean ± s.d. of at least three experiments reported as percentage accumulation of BCECF compared with the appropriate wild type (WT) control (without indomethacin). Accumulation of BCECF was assayed by fluorescence measurements, performed as described in Materials and methods. + and – symbols indicate the presence or absence of indomethacin (10 μM). (**A**) Murine cell lines: PC4-WT (WT), PC-V40 (V40) and PC-V160 (V160). (**B**) Human cell lines: HL60-WT (WT), HL60/ADR (ADR) and HL60/Vinc (Vinc)

washed in PBS, collected by centrifugation and resuspended in PBS–glucose with or without 10 μM indomethacin and placed in a shaking water bath at 37°C. Samples, taken at the indicated time points, were centrifuged to collect the cells and kept on ice before assaying by fluorescence spectroscopy for the amount of dye retained, as described above.

### Drug susceptibility studies

Sensitivity of each cell line to the chemotherapeutic agents, vincristine, doxorubicin or etoposide, was determined using the 48-h MTT colorimetric assay (Mosmann, 1983; Slapak et al, 1990). Briefly, 1 ml of $2 \times 10^4$ cells, with or without indomethacin (10 μM), were seeded into a 24-well plate containing increasing concentrations of the chemotherapeutic agent. After a 48-h incubation, cells were assayed for viability by the MTT assay. The $IC_{50}$ (dose of drug which reduced the final absorbance to 50% of control) was read from the dose–response curves.

### Vincristine steady-state accumulation studies

Vincristine (VCR) accumulation was assayed in $10^6$ cells $ml^{-1}$ after a 60 min incubation at 37°C in PBS–glucose (1 mg $ml^{-1}$) using 25 nM [G-$^3$H]vincristine sulphate (6.6–8.6 Ci $mmol^{-1}$, Amersham, Arlington Heights, IL, USA) in the absence or presence of indomethacin (10 μM). Cell-associated radioactivity was determined after centrifugation of samples ($2 \times 10^5$ cells) through silicone oil in microfuges tubes previously prepared with 20 *ul* of formic acid overlaid with 200 μl of silicone oil (density = 1.035–1.046, Nye Lubricants, New Bedford, MA USA). After freezing the tubes, the tips were severed (containing the frozen formic acid/cell pellet) and the cell-associated radioactivity was determined by scintillation counting.

### RESULTS

### Effect of indomethacin on BCECF-AM accumulation in murine or human cells expressing MRP and/or P-gp

We have previously shown that multidrug-resistant human and murine cell lines expressing the MRP have a decreased accumulation of the fluorescent dye BCECF, reflecting increased dye efflux (Draper et al, 1996). We examined the effect of various compounds on BCECF accumulation in MRP-overexpressing murine (PC4) (Slapak et al, 1994) and human (HL60) (Krishnamachary et al, 1994) leukaemia cell lines. One such compound, indomethacin (10 μM), caused a dramatic increase in the accumulation of BCECF in both the murine (PC-V40) and the human (HL60/ADR) MRP-expressing cell lines, normalizing drug accumulation to wild type levels (Figure 1). The murine PC4-WT and PC-V160 (overexpressing both P-gp and MRP) (Slapak et al, 1994) also showed an increase in BCECF accumulation, however, neither HL60WT nor HL60/Vinc (a P-gp-overexpressing cell line) (McGrath et al, 1989) showed an increase in BCECF accumulation. This pattern suggested activity against cells expressing MRP. The indomethacin-mediated increase in BCECF accumulation in PC4-WT could be the result of the low levels of MRP expressed in these cells (Slapak et al, 1994). Indomethacin appeared to have a greater effect on the MRP-overexpressing murine cells than on the human cell lines. Use of indomethacin at concentrations less than 1 μM caused little change in BCECF accumulation (data not shown).

A 1-h incubation of cells (PC4-WT, PC4-V40 and PC4-V160) with 10 μM indomethacin did not detectably alter the intracellular glutathione (GSH) content (e.g. PC4-V40; control = 2.5 ± 0.15 nmol $10^{-6}$ cells, indomethacin = 2.4 ± 0.25 nmol $10^{-6}$ cells). Indomethacin is a potent inhibitor of cyclo-oxygenase activity.

© Cancer Research Campaign 1997 

**Figure 2** Effect of indomethacin on the efflux of BCECF. Efflux experiments were performed as described in Materials and methods. Data are the mean of three experiments and are expressed as a percentage of BCECF present at time zero. By paired *t*-tests, the values for PC-V40 (+) indomethacin versus (–) indomethacin at the 20-min time point were significant at $P \leq 0.05$. + and – indicate the presence or absence of indomethacin (10 μM)

**Figure 3** Rhodamine accumulation in murine (**A**) and human (**B**) cell lines expressing MRP and/or P-gp. Accumulation of rhodamine was performed as described in Materials and methods. Data are the mean ± s.d. of at least three experiments. Cells were incubated without (–) or with (+) 10 μM indomethacin. Results are presented as percentage accumulation compared with the appropriate wild type control in the absence of indomethacin. (**A**) PC-WT (WT), PC-V40 (V40) and PC-V160 (V160); (**B**) HL60-WT (WT), HL60/ADR (ADR) and HL60/Vinc (Vinc)

However, inhibition of cyclo-oxygenase did not appear to be the mechanism behind the modulation of BCECF accumulation as the addition of exogenous prostaglandins (0.1 μM PGE1 or PGE2) did not detectably modify the altered BCECF accumulation in the MRP-overexpressing cell lines, nor did they ameliorate the effect of 10 μM indomethacin on BCECF accumulation in these cell lines (data not shown).

### Effect of indomethacin on efflux of BCECF by multidrug-resistant murine cell lines

We examined whether the indomethacin-mediated increased accumulation of BCECF was due to inhibition of dye efflux. Murine cell lines were loaded with BCECF in the presence of 10 μM indomethacin which normalized the dye loading among the cell lines. Cells were then washed in PBS–glucose to remove any extracellular BCECF-AM derivative or indomethacin and allowed to incubate in BCECF-AM-free PBS–glucose at 37°C in the presence or absence of indomethacin (10 μM). At various times, samples were taken to determine the dye levels in the cells. The presence of indomethacin inhibited the efflux of dye from the PC-V40 cell line (Figure 2A). A small effect was noted in PC-V160 which overexpresses MRP as well as P-gp (Slapak et al, 1994). These findings indicate that the increased accumulation of BCECF in cells incubated with indomethacin likely occurred via inhibition of efflux of the free-acid form of BCECF.

© Cancer Research Campaign 1997

**Table 1** Effect of indomethacin on drug susceptibility in murine and human cell lines

| Cell line (ABC transporter) | $IC_{50}$[a] Dox[b] (fold resistance)[c] Control | +Indomethacin | VCR[b] (fold resistance)[c] Control | +Indomethacin |
|---|---|---|---|---|
| *Murine* | | | | |
| PC4-WT | ND | ND | 2.5 ± 0.26 (1) | 1.6 ± 0.35 (0.64) |
| PC-V40 (MRP) | ND | ND | 167 ± 17 (67) | 31 ± 9.5 (12) |
| PC-V160 (MRP and P-gp) | ND | ND | 330 ± 60 (132) | 350 ± 87 (140) |
| PC4-80 (P-gp)[d] | ND | ND | 78 ± 7 (31) | 102 ± 39 (41) |
| *Human* | | | | |
| HL60WT | 43 ± 5.7 (1) | 36 ± 5.7 (.93) | 1.03 ± 0.03 (1) | 0.63 ± 0.08 (.61) |
| HL60/ADR (MRP) | 4333 ± 570 (100) | 1000 ± 115 (25) | 22 ± 2 (21) | 2.8 ± 0.7 (2.7) |
| HL60/Vinc (P-gp) | ND | ND | 900 ± 60 (873) | 950 ± 70 (922) |

[a]$IC_{50}$ (ng ml$^{-1}$) as determined by 48-h MTT assay. Data are the mean ± s.d. of three experiments. [b]DOX, doxorubicin; VCR, vincristine. [c]Fold resistance compared with wild type cells assayed without indomethacin. Fold resistance was calculated by dividing the mean $IC_{50}$ value by that for the parental cells (PC4-WT or HL60-WT). [d]Doxrubicin-selected P-gp-expressing cell line (Slapak et al, 1994). ND, not determined.

**Figure 4** Effect of indomethacin on vincristine accumulation in murine erythroleukaemia cell lines overexpressing the MRP and/or P-gp. Data are the mean ± s.d. of at least three experiments reported as fmol of drug 10$^{-6}$ cells. Accumulation of vincristine was assayed as described in Materials and methods. + and – symbols indicate the presence or absence of indomethacin (10 μM). Murine cell lines: PC4-WT (WT), PC-V40 (MRP overexpressing) (V40), PC-V160 (MRP and P-gp overexpressing) (V160) and PC4-80 (P-gp overexpressing) (A80)

### Effect of indomethacin on rhodamine transport

Rhodamine 123 has previously been shown to be a substrate for transport by P-gp (Tapiero et al, 1984). We examined the ability of cells expressing MRP or P-gp to accumulate rhodamine. As expected, the P-gp-expressing murine (PC-V160) and human (HL60/Vinc) cell lines showed a markedly decreased accumulation of rhodamine (Figure 3A and B). In contrast, the MRP-expressing lines (PC-V40 and HL60/ADR) showed no altered rhodamine accumulation compared with parental cells and, in fact, exhibited a slight increase in drug accumulation. This is in accord with previous results showing no altered accumulation of rhodamine, within the first 30 min of uptake, between parental and MRP-expressing cell lines (Twentyman et al, 1994). We then examined the effect of indomethacin on rhodamine accumulation. Indomethacin did not affect the accumulation of rhodamine by either MRP- or the P-gp-overexpressing cell lines compared with controls (Figure 3).

### Effect of indomethacin on drug susceptibility of murine and human cell lines

We examined the ability of indomethacin to function as a chemosensitizing agent. As determined by the MTT assay, the $IC_{10}$ for indomethacin was 50 μM for the wild-type and resistant murine cell lines and 20 μM for all the human cell lines. At a concentration of 10 μM, no toxicity was noted. In a 48-h MTT cell viability assay, 10 μM indomethacin reversed MRP-mediated vincristine resistance in both the murine and human cell lines (Table 1). As little doxorubicin resistance is expressed by the murine PC-V40, we examined the effect of indomethacin on doxorubicin resistance in the HL60/ADR cell line which overexpresses MRP and was originally established in doxorubicin. Against HL60/ADR indomethacin produced a dramatic chemosensitization to doxorubicin (Table 1). Indomethacin had no effect on P-gp-mediated vincristine resistance, further suggesting its specificity as a MRP modulator (Table 1). Of note, indomethacin had no effect on PC-V160 (expressing both MRP and P-gp) which is in accord with its previously noted P-gp-dominant phenotype (Figure 3) (Draper et al, 1996).

### Effect of indomethacin on vincristine accumulation

We evaluated the effect of indomethacin on a 60-min vincristine accumulation in the murine PC4-WT, PC-V40 (MRP expressing), PC-V160 (MRP and P-gp expressing) and the doxorubicin-selected P-gp-expressing PC4-80 cell lines. Indomethacin modulated the altered vincristine accumulation only in the PC-V40 (MRP expressing) cell line (Figure 4). Slight increases in vincristine accumulation were noted in the other cell lines, consistent with the BCECF accumulation data (Figure 1). This slight increase could be due to the effect of indomethacin on the low basal level of the MRP found in these cells.

### DISCUSSION

These studies show a modulation of drug transport in human and murine cells expressing MRP by indomethacin, a non-steroidal anti-inflammatory drug. Few reports describe compounds that specifically influence MRP-mediated multidrug resistance. One study, using the tyrosine kinase inhibitor genistein, demonstrated the modulation of daunorubicin accumulation in human small-cell lung cancer cells. The high concentration of genistein (200 μM) prohibited the use of this compound in a cell-proliferation assay (Versantvoort et al, 1994). Recently, Gekeler et al, have shown that MRP-associated resistance is efficiently modulated by the bisindolylmaleimide PKC inhibitor GF 109203X and the $LTD_4$-receptor antagonist MK571 (Gekeler et al, 1995*a,b*). In both cases, modulation of MRP-mediated resistance was shown using the MTT assay.

Depletion of cellular glutathione (GSH) levels with DL-buthionine (S,R)-sulphoximine (BSO), an inhibitor of γ-glutamyl cysteine synthetase, has been shown to modulate MRP-mediated resistance (Versantvoort et al, 1995; Zaman et al, 1995). The chemosensitizing effects of BSO are presumed to be due to the cellular depletion of GSH. In the vincristine-selected cell lines, we have demonstrated a reversal of vincristine and etoposide resistance by 25 μM BSO (Draper et al, 1996). However, the transport of some molecules, such as BCECF and calcein, is not affected by GSH depletion (Draper et al, 1996; Feller et al, 1995). The reason for the chemosensitization in response to depletion of intracellular GSH is unknown. It seems unlikely that the drugs are being transported as GSH-modified adducts, as no modified drugs can be detected (Zaman et al, 1995). This leaves open the possibility that drug transport is directly affected by GSH or that the transport of GSH or other GSH-modified compounds acts in some manner to facilitate transport of the chemotherapeutic agents.

In this present study, we have used the altered accumulation of the fluorescent dye BCECF to identify a modulator of multidrug resistance in MRP-overexpressing cell lines. Indomethacin both increased the accumulation of BCECF (Figure 1) and vincristine (Figure 4) and sensitized MRP-overexpressing cells to vincristine and doxorubicin (Table 1). It appears to have little effect on the function of P-gp as it failed to modulate P-gp-mediated altered accumulation of rhodamine (Figure 3) and vincristine (Figure 4) and failed to alter the drug resistance of both human and murine P-gp-overexpressing cell lines (Table 1). These data suggest that indomethacin is a specific modulator of MRP.

Indomethacin is a well-known inhibitor of prostaglandin synthesis and has also been shown to be a potent inhibitor of glutathione-S-transferase (Primiano and Novak, 1993). It has previously been used to enhance the anti-cancer activity of chlorambucil (Hall et al, 1989; Yang et al, 1992) and has also been shown to give partial reversal of methotrexate and cholate efflux in L1210 cells (Henderson et al, 1994).

The mechanism behind indomethacin's ability to chemosensitize MRP-overexpressing cells remains unknown at this time. As the addition of exogenous prostaglandins did not restore MRP function, it seems unlikely that indomethacin is functioning by inhibiting prostaglandin synthesis. This is in agreement with previous studies which looked at the effect of indomethacin on the efflux of BCECF in non-drug-resistant human and canine cell lines (Collington et al, 1991, 1992). Indomethacin also does not appear to be functioning by altering cellular GSH content as we could find no ability of the drug to alter GSH levels. There are two likely explanations for indomethacin function. First indomethacin could be interacting directly with MRP, functioning as a competitive inhibitor. Indomethacin has a pKa of 4.1, so that at physiological pH the drug would be in an anionic form (Tonnessen et al, 1989). MRP is known to display preference for more hydrophilic compounds and has been shown to transport amphiphilic organic anions (Cole et al, 1994; Leier et al, 1994). Its ability to block BCECF efflux suggests such an activity. However, as the MRP- and P-gp-expressing cell lines displayed no difference in the toxicity level of indomethacin, it does not seem likely that the drug is being effluxed; however, indomethacin may be binding to MRP and inhibiting efflux in another manner. Alternatively indomethacin may be functioning by inhibiting the function of GST, which may, like GSH, be necessary for proper drug efflux. Indomethacin has been shown to function as an inhibitor of class μ glutathione-S-transferases at a concentration of 1 μM (Primiano and Novak, 1993). Further, in a chlorambucil-resistant cell line demonstrating a 40-fold increase in an alpha class GST, indomethacin functioned as a chemosensitizing agent (Hall et al, 1989).

The clinical relevance of MRP is presently unknown. However, increases in P-gp and MRP have been reported in different types of leukaemias refractory to chemotherapy (Goasguen et al, 1993; Zhou et al, 1995). The concentration of indomethacin needed to increase drug susceptibility is clinically relevant (10 μM) and indomethacin is known to be well tolerated (Tonnessen et al, 1989; Statkevich et al, 1993). This suggests that indomethacin may be a valuable adjunct to chemotherapy to block MRP-mediated resistance as well as, in conjunction with a P-gp modulator, to deal with cells expressing both efflux proteins.

### ACKNOWLEDGEMENTS

This work was supported in part by USPHS grant CA59341 (SBL), the National Leukemia Research Association (SBL), the Zita Spiss Foundation (SBL), NRSA CA68800-01 (MPD) and USPHS Award J-32-CA09429 (RLM).

### ABBREVIATIONS

MRP, multidrug resistance protein; P-gp, P-glycoprotein; BCECF-AM, the fluorescent dye 2′,7′-bis-(2-carboxyethyl)-5-(6)-carboxyfluorescein acetoxymethylester; NSAID, non-steroidal anti-inflammatory drug.

### REFERENCES

Cole SPC, Sparks KE, Fraser K, Loe DW, Grant CE, Wilson GM and Deeley RG (1994) Pharmacological characterization of multidrug resistant MRP-

© Cancer Research Campaign 1997

transfected human tumor cells. *Cancer Res* **54:** 5902–5910

Collington GK, Allen CN, Simmons NL and Hirst BH (1991) Pharmacological profile of inhibition of 2′, 7′-bis(2-carboxyethyl)-5(6)-carboxyfluorescein efflux in human HCT-8 intestinal epithelial cells. *Biochem Pharmacol* **42:** S33–S38

Collington GK, Hunter J, Allen CN, Simmons NL and Hirst BH (1992) Polarized efflux of 2′,7′-bis(2-carboxyethyl)-5(6)-carboxyfluorescein from cultured epithelial cell monolayers. *Biochem Pharmacol* **44:** 417–424

Draper MP, Martell RL and Levy SB Active efflux of the free acid form of the fluorescent dye BCECF in MRP overexpressing murine and human leukemia cells. *Eur J Biochem* (In Press)

Feller N, Broxterman HJ, Währer DCR and Pinedo HM (1995) ATP-dependent efflux of calcein by the multidrug resistance protein (MRP): no inhibition by intracellular glutathione depletion. *FEBS* **368:** 385–388

Gekeler V, Boer R, Ise W, Sanders KH, Schachtele C and Beck J (1995*a*) The specific bisindolylmaleimide PKC-inhibitor GF 109 203X efficiently modulates MRP-associated multiple drug resistance. *Biochem Biophys Res Comm* **206:** 119–126

Gekeler V, Ise W, Sanders KH, Ulrich W-R and Beck J (1995*b*) The leukotriene $LTD_4$ receptor antagonist MK571 specifically modulates MRP associated multidrug resistance. *Biochem Biophys Res Comm* **208:** 345–352

Goasguen JE, Dossot J-M, Fardel O, LeMee F, LeGall E, Leblay R, RePrise PY, Chaperon J and Fauchet R (1993) Expression of the multidrug resistance-associated P-glycoprotein (P-170) in 59 cases of *de novo* acute lymphoblastic leukemia: prognostic implications. *Blood* **81:** 2394

Hall A, Robson CN, Hickson ID, Harris AL, Proctor SJ and Cattan AR (1989) Possible role of inhibition of glutathione *S*-transferase in the partial reversal of chlorambucil resistance by indomethacin in a Chinese hamster ovary cell line. *Cancer Res* **49:** 6265–6268

Henderson GB, Hughes TR and Saxena M (1994) Functional implications from the effects of 1-chloro-2,4-dinitrobenzene and ethacrynic acid on efflux routes for methotrexate and cholate in L1210 cells. *J Biol Chem* **269:** 13382–13389

Inaba M, Kobayashi H, Sakura Y and Johnson RK (1979) Active efflux of daunorubicin and adriamycin in sensitive and resistant sublines of P388 leukemia. *Cancer Res* **39:** 2200–2203

Krishnamachary N, Ma L, Zheng L, Safa AR and Center MS (1994) Analysis of MRP gene expression and function in HL60 cells isolated for resistance to adriamycin. *Oncol Res* **6:** 119–127

Leier I, Jedlitschky G, Buchholz U, Cole SPC, Deeley RG and Keppler D (1994) The *MRP* gene encodes an ATP-dependent export pump for leukotriene C4 and structurally related conjugates. *J Biol Chem* **269:** 27807–27810

McGrath T, Latoud C, Arnold ST, Safa AR and Felsted RL (1989) Mechanisms of multidrug resistance in HL60 cells. Analysis of resistance associated membrane proteins and levels of mdr gene expression. *Biochem Pharmacol* **38:** 3611–3619

Mosmann T. (1983) Rapid colorimetric assay for cellular growth and survival: application to proliferation and cytotoxicity assays. *J Immunol Methods* **65:** 55–63

Primiano T and Novak RF (1993) Purification and characterization of class μ glutathione S-transferase isozymes from rabbit hepatic tissue. *Arch Biochem Biophys* **301:** 404–410

Slapak CA, Daniel JC and Levy SB (1990) Sequential emergence of distinct resistance phenotypes in murine erythroleukemia cells under adriamycin selection: decreased anthracycline uptake precedes increased P-glycoprotein expression. *Cancer Res* **50:** 7895–7901

Slapak CA, Fracasso PM, Martell RL, Toppmeyer DL, Lecerf J-M and Levy SB (1994) Overexpression of the multidrug resistance-associated protein (MRP) gene in vincristine but not doxorubicin-selected multidrug-resistant murine erythroleukemia cells. *Cancer Res* **54:** 5607–5613

Statkevich P, Fournier DJ and Sweeney KR (1993) Characterization of methotrexate elimination and interaction with indomethacin and flurbiprofen in the isolated perfused rat kidney. *J Pharmacol Exp Ther* **265:** 1118–1124

Tapiero H, Munck J-C, Fourcade A and Lampidis T J (1984) Cross resistance to rhodamine 123 in adriamycin and daunorubicin-resistant Friend leukemia cell variants. *Cancer Res* **44:** 5544–5549

Tonnessen TI, Aas AT, Sandvig K and Olsnes S (1989) Inhibition of chloride/bicarbonate antiports in monkey kidney cells (Vero) by non-steroidal anti-inflammatory drugs. *Biochem Pharmacol* **38:** 3583–3591

Twentyman PR, Rhodes T and Rayner S (1994) A comparison of rhodamine 123 accumulation and efflux in cells with P-glycoprotein-mediated and MRP-associated multidrug resistance phenotypes. *Eur J Cancer* **30A:** 1360–1369

Versantvoort CHM, Broxterman HJ, Lankelma J, Feller N and Pinedo HM (1994) Competitive inhibition by genistein and ATP dependence of daunorubicin transport in intact MRP overexpressing human small cell lung cancer cells. *Biochem Pharmacol* **48:** 1129–1136

Versantvoort CHM, Broxterman HJ, Bagrij T, Scheper RJ and Twentyman PR (1995) Regulation by glutathione of drug transport in multidrug-resistant human lung tumour cell lines overexpressing multidrug resistance-associated protein. *Br J Cancer* **72:** 82–89

Yang WZ, Begleiter A, Johnston JB, Israels LG and Mowat MRA (1992) Role of glutathione and glutathione *S*-transferase in chlorambucil resistance. *Molec Pharmacol* **41:** 625–630

Zaman GJR, Flens MJ, van Leusden MR, de Haas M, Mülder HS, Lankelma J, Pinedo HM, Scheper RJ, Baas F, Broxterman HJ and Borst P and (1994) The human multidrug resistance-associated protein MRP is a plasma membrane drug-efflux pump. *Proc Natl Acad Sci USA* **91:** 8822–8826

Zaman GJR, Lankelma J, van Tellingen O, Beijnen J, Dekker H, Paulusma C, Elferink RPJ O, Baas F and Borst P. (1995) Role of glutathione in the export of compounds from cells by the multidrug-resistance-associated protein. *Proc Natl Acad Sci USA* **92:** 7690–7694

Zhou DC, Zittour R and Marie JP (1995) Expression of multidrug resistance-associated protein (MRP) and multidrug resistance (MDR1) genes in acute myeloid leukemia. *Leukemia* **9:** 1661–1666

© Cancer Research Campaign 1997